# Unusual leonine facies: A rare presentation

**DOI:** 10.1002/kjm2.12881

**Published:** 2024-07-29

**Authors:** Jiong‐Huang Lim, Feng‐Ling Lin

**Affiliations:** ^1^ Department of Dermatology Cathay General Hospital Taipei Taiwan

Leonine facies is an uncommon morphological presentation produced by thickening, furrowing, and coarsening of the facial skin. Various conditions are associated with its occurrence, including lepromatous leprosy. Herein, we describe the case of a woman who developed leonine facies secondary to presenile diffuse familial sebaceous gland hyperplasia (PDFSH).

A 37‐year‐old healthy woman from Indonesia visited our clinic with a 3‐year history of developing facial lesions without any other symptoms. Her father had a similar skin condition. She reported no loss of sensation (hypoesthesia) in the affected areas. Upon physical examination, we observed multiple hardened papules and plaques on her face forming grooves and fissures. The lesions spared the areas around the mouth, nose, ears, and eyes. The eyebrows were intact (Figure 1A). Lepromatous leprosy was considered on the basis of the skin findings. However, a biopsy of the affected tissues revealed typical sebaceous hyperplasia (Figure 1C, D). Negative results from acid‐fast staining ruled out leprosy as a diagnosis. The absence of granulomatous inflammation, mucin, neoplastic cells, fibroblast proliferation, and fibrosis excluded other potential diagnoses such as sarcoidosis, leishmaniasis, and cutaneous T‐cell lymphoma. The patient's clinical and pathological findings ruled out lepromatous leprosy, and PDFSH was determined as the likely diagnosis. The patient was prescribed isotretinoin; she took 10 mg daily and experienced substantial improvement after 1 month (Figure 1B).

**FIGURE 1:**
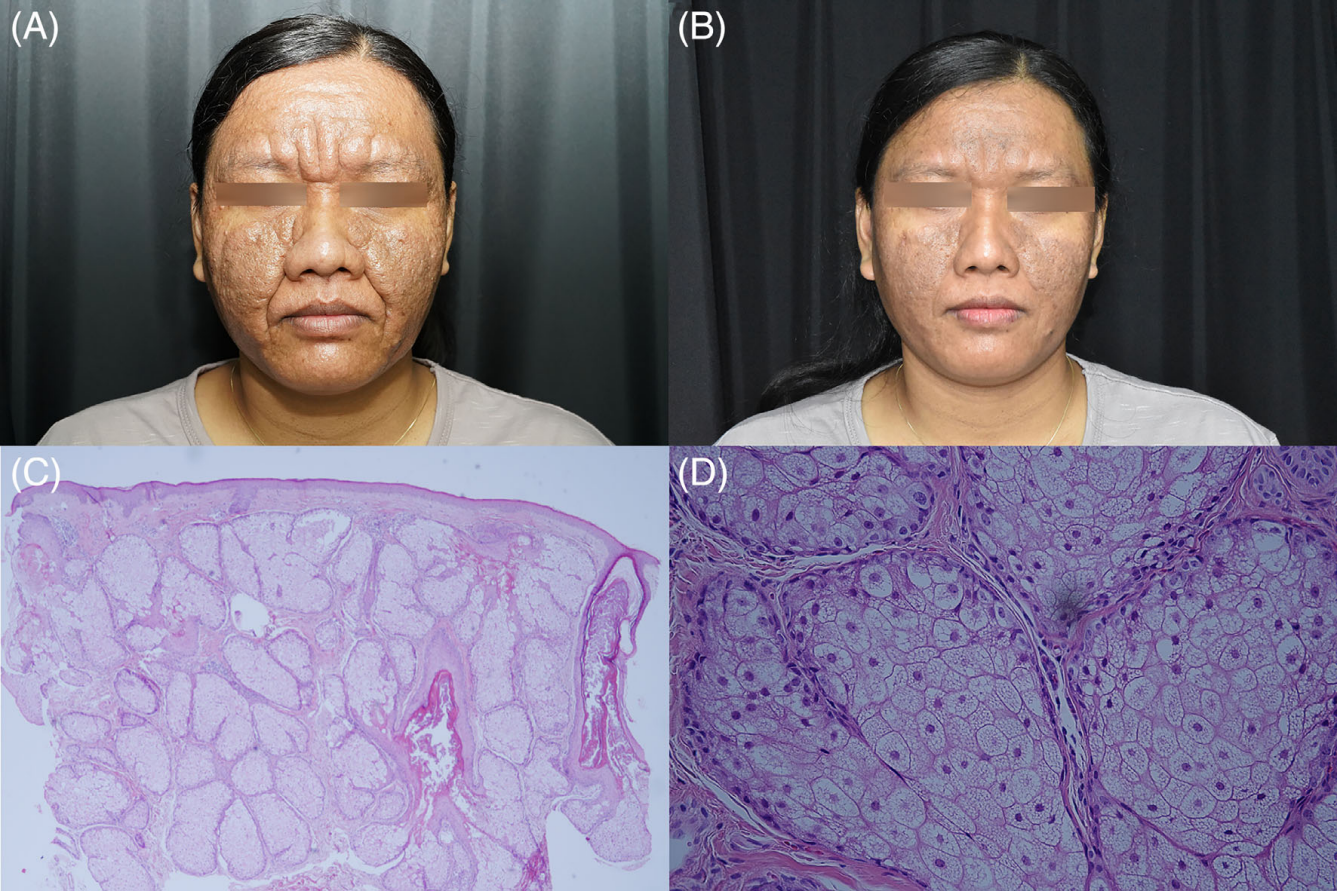
(A) Clinical presentation revealing multiple hardened, skin‐colored to brownish papules and facial plaques sparing the periorbital, perinasal, and perioral areas and the auricular regions. (B) The patient at 1 month after being prescribed 10 mg daily of isotretinoin. (C, D) Histopathology with expanded lobules of sebaceous glands without thickening of peripheral generative cells (C: Hematoxylin–eosin ×40, D: Hematoxylin–eosin ×400).

Additional conditions associated with leonine facies include cutaneous sarcoidosis, leishmaniasis, chronic dermatologic disorders, and hematological malignancies.^1^ A clinical manifestation of PDFSH with leonine facies, such as that in the present report, is far less common.

PDFSH is diagnosed on the basis of clinical features and family history. A biopsy may be performed to confirm the diagnosis, especially in cases of uncertainty or suspicion of other conditions. PDFSH is thought to be a genetic disorder that affects the sebaceous glands in the skin, causing overgrowth of these glands and the formation of small, yellowish bumps on the skin. Typically, PDFSH manifests during puberty or shortly thereafter and progresses gradually. The bumps related to this condition are not painful and do not elicit any other symptoms. Notably, the periorificial regions, such as the periorbital, perinasal, preauricular, and perioral areas, remain unaffected. The histopathology of PDFSH is indistinguishable from its senile counterpart, with both characterized by enlarged sebaceous glands containing fully developed sebaceous lobules that drain into an expanded central sebaceous duct.^2^ Isotretinoin's antiproliferative properties result in a noticeable reduction in sebaceous gland size when used to treat these conditions. A daily dose of 10–25 mg typically results in rapid symptom relief.^2^, ^3^, ^4^ In the present case, the patient experienced a dramatic improvement in symptoms after being prescribed a daily dose of 10 mg for 1 month.

Lepromatous leprosy is caused by the Mycobacterium leprae complex and is characterized by slow‐growing, obligate intracellular, acid‐fast bacilli. Leprosy is prevalent in low‐ and middle‐income countries, particularly India, Indonesia, and Brazil. Lepromatous leprosy causes lesions on the face, scalp, fingers, and toes, whereas warmer body parts generally remain unaffected. As the disease progresses, facial infiltration may result in deeper forehead furrows, nasal mucosa involvement, leonine facies, and eyebrow loss. A biopsy typically reveals an atrophic epidermis and Grenz zones characterized by a band of dermis with a normal appearance that separates the epidermis from the infiltrate. Foamy macrophages, scattered plasma cells, and lymphocytes are distributed in the dermis. A perineural collection of macrophages surrounding the “onion skin” of the perineurium is typically present. Acid‐fast bacilli are often clumped in these macrophages, forming clusters or globi.^5^

In conclusion, we presented a rare case in which PDFSH manifested as leonine facies. This presentation must be carefully distinguished from that of lepromatous leprosy. Administering a 10‐mg daily dose of isotretinoin is a safe and efficient method for managing PDFSH.

## CONFLICT OF INTEREST STATEMENT

The authors declare no conflicts of interest.

## INFORMED CONSENT

The patient has provided informed consent for publication of the case.

## References

[1] Ghahartars M , Parvar SY , Samipour L , Hadibarhaghtalab M . Trichoepithelioma presenting as leonine facies in a young female. Skin Health Dis. 2022;3(1):e177. 36751311 10.1002/ski2.177 PMC9892421

[2] Liu YS , Cheng YP , Liu CI , Yang CY , Yang CY . Presenile diffuse familial sebaceous hyperplasia successfully treated with low‐dose isotretinoin: a report of two cases and review of the published work. J Dermatol. 2016;43(10):1205–1208. 27130181 10.1111/1346-8138.13416

[3] Abdel‐Halim MRE , El‐Nabarawy E , El‐Tawdy A , Fawzy MM , Shalaby S , Ismail S , et al. Leonine facies and neck papules. Int J Dermatol. 2019;58(7):797–799. 30839101 10.1111/ijd.14428

[4] Low QJ , Cheo SW , Yap WYE . Leonine facies. Indian J Dermatol Venereol Leprol. 2021;87(4):589–591. 32068196 10.4103/ijdvl.IJDVL_983_18

[5] Maymone MBC , Laughter M , Venkatesh S , Dacso MM , Rao PN , Stryjewska BM , et al. Leprosy: clinical aspects and diagnostic techniques. J Am Acad Dermatol. 2020;83(1):1–14. 32229279 10.1016/j.jaad.2019.12.080

